# Fuzzy-Based Trust Prediction Model for Routing in WSNs

**DOI:** 10.1155/2014/480202

**Published:** 2014-07-14

**Authors:** X. Anita, M. A. Bhagyaveni, J. Martin Leo Manickam

**Affiliations:** ^1^Department of ECE, Anna University, Chennai 600025, India; ^2^Department of ECE, St. Joseph's College of Engineering, Chennai 600119, India

## Abstract

The cooperative nature of multihop wireless sensor networks (WSNs) makes it vulnerable to varied types of attacks. The sensitive application environments and resource constraints of WSNs mandate the requirement of lightweight security scheme. The earlier security solutions were based on historical behavior of neighbor but the security can be enhanced by predicting the future behavior of the nodes in the network. In this paper, we proposed a fuzzy-based trust prediction model for routing (FTPR) in WSNs with minimal overhead in regard to memory and energy consumption. FTPR incorporates a trust prediction model that predicts the future behavior of the neighbor based on the historical behavior, fluctuations in trust value over a period of time, and recommendation inconsistency. In order to reduce the control overhead, FTPR received recommendations from a subset of neighbors who had maximum number of interactions with the requestor. Theoretical analysis and simulation results of FTPR protocol demonstrate higher packet delivery ratio, higher network lifetime, lower end-to-end delay, and lower memory and energy consumption than the traditional and existing trust-based routing schemes.

## 1. Introduction

Wireless sensor networks (WSNs) have attractive wider range of application from civil sector to military [1–5]. A WSN consists of large number of resource constraint sensor nodes (SNs) deployed in hostile environment which makes it feasible for adversaries to perform varied types of attack [6, 7]. Due to the limited communication range [8], the SNs communicate with the sink in multihop. This cooperative nature of WSNs makes it vulnerable to insider attack which requires a trust management scheme. Most of the trust management schemes proposed in the literature were dependent on direct and indirect observations.

For direct trust computations, the promiscuous mode of operation was used in most of the trust-based routing protocols for neighbor monitoring to compute direct trust. It demands that nodes should be in wakeup state for longer duration which incurs more energy. The indirect trust was computed by receiving recommendation from all the neighbors which also consumes more energy. Moreover, the malicious nodes were identified based on their historical trust. Hence, in order to reduce the damages caused due to malicious activities in mission critical applications, the behavior of a node should be predicted in advance based on the historical trust and the tendency of that node to maintain it consistently with the neighboring nodes in the network.

To address these issues, in this paper we proposed a novel fuzzy-based trust prediction model for routing (FTPR) in WSNs. FTPR was designed with the following objectives:

- To minimize the energy consumption by avoiding promiscuous mode of operation for neighbor monitoring and by reducing the number of recommendations collected from neighbors to compute indirect trust.
- To reduce packet loss by identifying and eliminating malicious nodes by trust prediction. The trust of a neighboring node is predicted based on direct trust, number of trust fluctuations, and recommendation inconsistency.
- To thwart black hole attack, on-off attack, bad-mouthing attack, and conflicting behavior.

This paper is organized as follows: Section 2 discusses the related work. In Section 3, we described the framework of the proposed FTPR protocol. Simulation results and theoretical analysis are discussed in Section 4, and Section 5 concludes the paper along with the future scope of the work.

## 2. Related Works

Several trust-based routing schemes proposed recently in the literature were designed not only to meet the security requirements but also considered the resource constraint nature of WSNs.

Paris et al. proposed a novel routing protocol to eliminate the selfish behavior of a neighbor [9]. The scheme used a novel routing metric called expected forwarding counter (EFW) that was used to thwart selective forwarding attack in wireless mesh networks. EFW was a cross-layer metric updated based on the observation of network layer and MAC layer. Mohi et al. proposed an intrusion detection scheme to eliminate denial-of-service (DoS) attack using Bayesian game approach in WSNs [10]. It was an incentive-based approach that motivates the nodes to behave properly. DoS attack was prevented based on the past behavior of the nodes in the Bayesian game formulation. Fuzzy-based detection and prediction system (FBDPS) [11] was proposed to detect distributed DoS (DDoS) attack. FBDPS compared the actual energy consumed by a neighbor with the normal value. When the energy consumed by that node was abnormal, then the node was considered as malicious. The drawback of these schemes was their ability to identify only a specific attack which may not be suitable for realistic applications.

Group-based trust management scheme (GTMS) [12] was designed to overcome black hole attack. The trust was dependent on direct and indirect monitoring. A distributed trust management scheme was adopted in intragroup level by collecting recommendations from all its group members to compute trust. A centralized trust management approach was used in intergroup level as each cluster head (CH) collected recommendations of other CHs directly from the sink. In order to reduce the memory consumption, the trust was represented as unsigned integers in the range from 0 to 100. The drawback of the GTMS was in the requirement of high energy CHs to directly communicate with the sink. Ambient trust sensor routing (ATSR) [13] was proposed to thwart black hole attack, bad-mouthing attack, and conflicting behavior. It was a geographic routing protocol and trust was computed based on direct and indirect observations. The trust values were represented as real numbers in the range from 0 to 1. Lightweight and dependable trust system (LDTS) [14] designed for hierarchical WSNs thwarted black hole and bad-mouthing attacks. The trust was computed based on direct and indirect observations. A centralized trust management scheme was used in intracluster and intercluster level. The trust value was assigned in the range of 0 to 10. All the above-mentioned schemes use promiscuous mode of operation for direct observation. The malicious nodes were identified only based on the past experience of a node.

In order to improve the network security through trust prediction, trust-based source routing protocol (TSR) [15] was proposed for mobile ad hoc networks (MANET). Fuzzy logic-based approach was used to predict the future behavior of a node from the knowledge of past behaviors. Trust was derived from the direct observations and TSR was able to thwart black hole attack and grey hole attack. Ad hoc on-demand trusted multipath distance vector routing protocol (AOTMDV) [16] was proposed for MANET to eliminate modification attack, black hole attack, and grey hole attack. It derived the trust based on direct and indirect observations. It used all the received recommendations to compute the historical trust of node which made it vulnerable to bad-mouthing attack. Trust-aware secure routing framework (TSRF) [8] proposed for WSNs was based on direct and indirect observations. It was designed to thwart grey hole, tampering, on-off and bad-mouthing attack. As the trust value was represented as real numbers in the range from 0 to 1, TSRF consumed more memory. The malicious nodes were identified only based on historical trust of a node. Two-way acknowledgment-based trust (2-ACKT) [17] framework did not use promiscuous mode of operation for trust derivation and thwarted the black hole attack in WSNs. It used acknowledgments to derive the trust on the neighboring nodes. The scheme assumed that the malicious node drops data packets alone and not the acknowledgments. The scheme depends only on direct trust. As the recommendations were not gathered from the neighboring nodes, the decisions derived might not be fully consistent with the actual state of the network.

## 3. Fuzzy-Based Trust Prediction Routing Protocol

In this section, we discussed the detailed framework of our proposed FTPR protocol for WSNs. The assumptions made for the protocol design and the threat model employed for evaluating the performance of the protocol were also presented. FTPR protocol derived the trust based on direct and indirect observations.

### 3.1. Assumptions and Threat Model

In WSNs, each node forwards the data to the sinks with the help of other intermediate nodes. The number of sinks did not have any impact on the FTPR protocol. Hence, for simplicity we assumed there was only one sink in the network. We assumed a hierarchical topology that consists of CHs and cluster members (CMs). The FTPR protocol maintains intracluster and intercluster topologies. The intragroup topology consists of group of CMs which were attached to a CH. Intergroup topology comprises CHs and sink. The proposed FTPR framework consisted of two stages, namely, route discovery stage and data forwarding stage. During route discovery stage, each node discovered a route to the sink using a routing protocol and it was assumed that all nodes behaved legitimately during this stage. In data forwarding stage, each CM forwarded the data to the CH and CH in turn forwarded the data to the sink using multihop communication link. We assumed that some of the intermediate nodes in the multihop communication link behaved maliciously while forwarding the data packets. Basically trust is a relationship associating between two nodes for a specific action. In FTPR, we derived the trust between any two communicating nodes based on packet forwarding action. An adversary can modify the contents of data packets or various control packets exchanged between the neighboring nodes in FTPR protocol. In order to prevent fabrication of control or data packets, a secure communication channel which can be established with the help of any key management schemes [18–21].

We assumed malicious nodes manifested black hole attack [8], on-off attack [8], bad-mouthing attack [8], and conflicting behavior attack [8]. The black hole attack and on-off attack are manifested in the data forwarding plane. In black hole attack, a malicious node drops all the received packets instead of forwarding. It behaves well and bad alternatively in on-off attack, hoping that it can remain undetected while misbehaving. The bad-mouthing attacks and conflicting behavior are manifested in the trust evaluation plane. In bad-mouthing attack, a malicious node provides dishonest feedback to recommend good node as bad node and bad node as good node. In conflicting behavior attack, a malicious node behaves differently to nodes in different groups.

### 3.2. Network Topology

**Figure 1:**
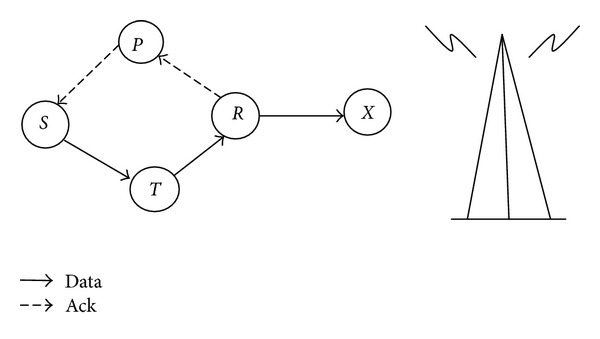
Network topology.

Consider the topology shown in Figure 1. Let node *S* be the subject wanted to evaluate the trust on its neighbor node target *T*. Node *S* forwards the data packet to its neighbor node *T* which in turn forwards the packet to its neighbor node sponsor *R*. On receiving the data packet, sponsor *R* forwards the data packet to its neighbor *X* as well as transmits an acknowledgment to node *S* through third party *P* as shown in the Figure 1. 2-ACKT protocol [17] was used for routing and determination of third party for the transmission of acknowledgment. A transaction was considered to be successful when the subject receives the acknowledgment for the data packet sent to target *T*. The higher the number of successful transactions, the higher the trust on the target.

In FTPR, the trust was computed in intracluster level and intercluster level. In intracluster level, the CH aggregates all the data packets transmitted by the CMs. Some of the intermediate CMs in the communication link were malicious nodes. In FTPR protocol, when a CM “x” wanted to communicate with the CH through the intermediate CM “y,” then node x would check the trust of node y in its trust table. If node y was legitimate, then node x would transmit the data packet to node y; otherwise node x would find another route to the CH. Within the cluster, the trust was based on direct observation only as discussed in Section 3.3. In order to reduce the overhead involved in gathering recommendations, the indirect trust was not considered at the intracluster level. In intercluster level, the CH sends all the aggregated data to the sink through the multihop communication link which may contain malicious nodes. In FTPR protocol, when a CH “x” wanted to communicate with the sink through the intermediate CH “y,” then node x would check the trust of CH “y” in its trust table. If the CH “y” was legitimate, then CH “x” would transmit the data packet to node y; otherwise node x would find another route to the sink. The trust was computed based on direct trust and indirect trust as discussed in Section 3.4. Indirect trust was considered to maintain trust consistency within the network.

### 3.3. Direct Trust Computation

In trust-based routing schemes, direct trust was calculated based on direct interaction with the neighbors. It must ensure that the neighbor had successfully received the packet and then forwarded the packet honestly by following the underlying routing protocol. The packet forwarding behavior of a CM was monitored by two-hop group acknowledgment scheme as discussed in [17].

**Figure 2:**
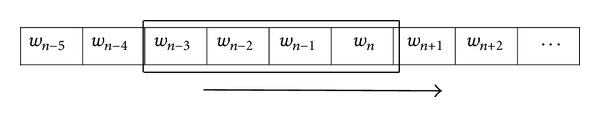
Sliding time window.

In order to identify the inconsistent behavior of a node, the historical trust of a node should be considered to compute trust. To address these issues, a sliding time window scheme for trust calculation was used as shown in Figure 2. The time scale was divided into equal sized observation windows such as *w*_*n*−5_, *w*_*n*−4_, *w*_*n*−3_, *w*_*n*−2_, *w*_*n*−1_, *w*_*n*_, *w*_*n*+1_,…, where *w*_*n*_ was the *n*th observation window. The numbers of successful and failed transactions were calculated for each observation window.

The sliding time window consisted of four observation windows as shown in Figure 2. The details of interactions in each observation window were recorded separately. Trust of a node was computed based on the numbers of successful and failed transactions. For each unit of time, the sliding time window slides one observation window to the right, thereby dropping the older experience by one unit and adds up the newer experience. Hence, the trust on the target during the *n*th observation window *w*_*n*_ depends on the numbers of successful and failed transactions during four observation windows, namely, *w*_*n*_, *w*_*n*−1_, *w*_*n*−2_, and *w*_*n*−3_. In order to store the details gained during direct interaction, we introduced a transaction table in the routing protocol. The observed successful and failed transactions were stored in the transaction table.

The transaction table of the CM consisted of the following fields:

 
*〈* *node id, number of successful transactions, number of failed transactions, trust level* *〉*,

where* node id* was the address of the target, the* number of successful transactions* field was incremented by one whenever it received a link-layer acknowledgment from the target and a third-party acknowledgment within a timeout period, the* number of failed transactions* was incremented by one whenever it received a link-layer acknowledgment from the target and not the third-party acknowledgment within a given timeout period, and* trust level* can take an integer value that lies in the range from 0 to 7. The computed trust value that lies in the range from 0 to 100 was mapped to a trust level which lies in range from 0 to 7 as discussed in [17].

As the CH computed trust based on direct observations as well as from the neighbor's recommendations, the transaction table of CH consists of the following fields.

 
*〈* *node id, number of recent transactions, number of successful transactions, number of failed transactions, trust level, peer recommendation, number of fluctuations, recommendation inconsistency, predicted trust* *〉*,

where* peer recommendation* field was updated based on the recommendations received from the neighbors,* number of recent transactions* was incremented by one when it received a data packet from the neighbor* node id*, and* number of fluctuations* was used to monitor frequency of change in the trust level of neighboring node. A good node maintains a constant trust level and hence the number of fluctuations was low. The number of fluctuations (*T*_NF_) can be updated as given by

(1) $$\begin{array}{c} T_{\text{NF}}=\{\begin{array}{cc} T_{\text{PNF}}-T_{\text{CTL}}+T_{\text{MTL}}, & T_{\text{CTL}}<T_{\text{PTL}},\\ T_{\text{PNF}}-1, & T_{\text{CTL}}\geq T_{\text{PTL}}, \end{array} \end{array}$$

where *T*_PNF_ was the previous number of fluctuations and *T*_MTL_ was the maximum trust level; *T*_PTL_ and *T*_CTL_were the previous and current trust level.

The equation was designed in such a way that *T*_NF_ increases rapidly when the trust level decreases.

The* recommendation inconsistency* was used to monitor whether the target was behaving in a consistent manner with all its neighbors and it is equal to the variance (*σ* ^2^) of the received recommendations. The variance of the recommendations received for a target that behaves uniformly with all its neighbors is lesser than for a target that behaves inconsistently with its neighbors. It was used to identify the conflicting behavior of node.

The* predicted trust *was updated using the fuzzy-based trust prediction model discussed in Section 3.6.

The direct trust between the subject *S* and target *T* based on the number of successful and failed transactions can be derived as follows. Let *T*_DV_(*S*, *T*) be the direct trust value of *T* computed by *S*. It was initially assumed to be 100 since all nodes were considered legitimate during network setup. Let *T*_*s*_*i*__ and *T*_*f*_*i*__ be the number of successful and failed transactions during the *i*th observation window, respectively; then

(2) $$\begin{array}{c} T_{\text{DV}}\left(S,T\right)=\left(\frac{\sum_{i=0}^{n-1}\alpha_{i}P_{i}\left(1-P_{i}\right)}{\sum_{i=0}^{n-1}\alpha_{i}\left(1-P_{i}\right)}\right)100, \end{array}$$

where *n* is the total number of observation windows and *P*_*i*_ represents positive trust value given by

(3) $$\begin{array}{c} P_{i}=\left(\frac{T_{s_{i}}+1}{T_{s_{i}}+T_{f_{i}}+2}\right)\left(1-\frac{1}{T_{s_{i}}+2}\right). \end{array}$$

The term (*T*_*s*_*i*__ + 1)/(*T*_*s*_*i*__ + *T*_*f*_*i*__ + 2) in (3) simply gives the ratio of number of successful transactions to the total number of transactions during the *i*th observation window. In order to give more importance to the number of successful transactions, the ratio (*T*_*s*_*i*__ + 1)/(*T*_*s*_*i*__ + *T*_*f*_*i*__ + 2) was multiplied by the term 1 − (1/(*T*_*s*_*i*__ + 2)). As the bad behavior of a node should be remembered for longer duration and the recent transactions must carry more significance than older transactions, *P*_*i*_ was multiplied by *α*_*i*_(1 − *P*_*i*_) while calculating the trust value, where *α*_*i*_ is an aging factor and 1 − *P*_*i*_ represents negative trust value for *i*th observation window. The value of *α*_*i*_ can take any values in the range from 0 to 1 subject to the condition that *α*_1_ < *α*_2_ < ⋯<*α*_*n*_.

#### 3.3.1. Trust Counselor

The two-hop acknowledgments depend on an alternate path through sponsor and third party. So, the trust of the target might be reduced due to the malicious activity of the sponsor or target or both. In order to identify the malicious activity of sponsor and target, a trust counselor component was introduced.

When the trust level of the target dropped below a warning threshold (*T*_*w*_), then the subject initiated the counseling process by unicasting a warning packet to the target. *T*_*w*_ was not a constant and it was determined based on the trust level of the trusted neighboring nodes. The warning packet comprises the following fields:

 
*〈* *warning identifier, subject address, packet category, node address_1, node address_2* *〉*,

where* warning identifier* and* subject address* uniquely identified the warning packet and* warning identifier* was defined by the subject;* packet category* was assigned 0 or 1 if the* node address_2* had not forwarded data or acknowledgment packets, respectively. It was assigned 0 when the subject initiated the counseling process as it assumed target had not forwarded the data packet. When the subject unicasted a warning packet to target,* node address_1* and* node address_2* were set as subject and target addresses, respectively.

On receiving the warning packet, target modified the packet category to 1 as it assumed that the sponsor had not forwarded the acknowledgment back to the subject through the alternate path. In this way the warning packet reached the third party through the sponsor. On receiving the warning packet, the third party unicasts a response packet back to the sponsor. The response packet consists of the following fields:

 
*〈* *response identifier, subject address, node address_1, node address_2, status* *〉*,

where* response identifier* and* subject address* were assigned with the* warning identifier* and* subject address* as mentioned in the warning packet, respectively.* node address_1* was assigned with its own address (third party address) and* node address_2* was assigned with the sponsor address. The status field can be 0, 1, 2, 3, 4, or 5 as mentioned in the Table 1.

Status 0 denoted link failure and not yet rectified. Hence, the node was not ready to forward any packet. Status 1 referred to the condition that the node had not forwarded the packet due to link failure; but later the failure was rectified and ready to forward. Link failure could be due to network traffic conditions. Status 2 implied the inability of a node to participate in data forwarding activity due to energy or bandwidth unavailability. Status 3 referred to the condition that the node had not forwarded the packets due to insufficient resources; but later the resources were available and ready to forward the packets. Status 4 was used to indicate the existence of noncooperative malicious neighbor and the unavailability of an alternate path. Status 5 was used when the node had identified another alternate path due to the existence of noncooperative neighbor.

In this way the response travelled back to the subject through the sponsor and target. If the response packet did not reach the subject within the response wait time, then the subject reinitiated the route discovery process. The time interval between the transmission of warning packet to the target and the reception of response generated by any one of the three entities, namely, target, sponsor, and third party is called response wait time. The upper bound for the response wait time is equal to the three times the sum of the propagation delay and the processing delay experienced by the packets in the network.

### 3.4. Indirect Trust Computation

The indirect trust for the target was computed based on the recommendations obtained from neighbors. Recommendations also help in building a trust consistent with the network. In this section, we discussed the procedures for requesting recommendations and responding to such requests in WSNs. For requesting recommendations, subject broadcasts a trust request (TREQ) message to its neighbors in the transmission range. The TREQ message contains the following fields:

 
*〈* *TReqId, subject, target, I*_min⁡_ *, timestamp* *〉*.

*TReqId *is the trust request identifier used to uniquely identify the TREQ message and* timestamp* indicated the issuing time. *I*_min⁡_ denotes the minimum number of interactions a recommender must have with the subject and it is given by

(4) $$\begin{array}{c} I_{\min}=\frac{1}{n}\overset{n}{\underset{i=0}{\sum }}T_{s_{i}}+T_{f_{i}}, \end{array}$$

where *n* is the number of neighboring CHs.

Algorithm 1(a) describes the procedure for transmitting the TREQ message in detail. Upon receiving the TREQ message, the nodes that had prior trust relationship with target processed the TREQ message as given in the Algorithm 1(b) and unicast trust reply (TREP) message back to the subject. The TREP message contains the following fields:

 
*〈* *recommender, subject, target, T*_DL_(*r*_*i*_, *T*)*〉*,

which indicates that a recommender (*r*_*i*_) unicasts the TREP message back to the subject which had a trust level of *T*_DL_(*r*_*i*_, *T*) with target.

Let us assume that *ψ* is the set of recommenders for the subject defined as

(5) $$\begin{array}{c} \psi =\left\{r_{i}:0\leq i\leq J-1\right\}, \end{array}$$

where *J* is the total number of recommenders. Then the indirect trust between the subject *S* and target *T* can be defined as

(6) $$\begin{array}{c} T_{\text{IL}}\left(S,T\right)=\frac{1}{J}\overset{J-1}{\underset{i=0}{\sum }}T_{\text{DL}}\left(r_{i},T\right), \end{array}$$

where *T*_DL_(*r*_*i*_, *T*) is the trust level associated between the *i*th recommender and the target *T*. It was an integer value represented in the range from 0 to 7. It can be noted that a malicious node can manifest bad-mouthing attacks while responding to the TREQ message.

In order to detect such outliers from the received recommendations, we used empirical rule [22] with mean (*μ*) plus or minus one standard deviation (*σ*) as the recommendations were represented as integer values in the range from 0 to 7. Only those recommendations that lie in the range from (*μ* − *σ*) to (*μ* + *σ*) were considered consistent and used for calculating *T*_IL_(*S*, *T*) from (6). Let us assume that there are *l* number of outliers in the *J* number of received recommendations, and then (6) can be rewritten as

(7) $$\begin{array}{c} T_{\text{IL}}\left(S,T\right)=\frac{1}{J-l}\overset{J-l-1}{\underset{i=0}{\sum }}T_{\text{DL}}\left(r_{i},T\right), \end{array}$$

where *l* ≤ *J*. A target was considered to be trusted when *T*_IL_(*S*, *T*) is greater than or equal to *T*_TL_.

### 3.5. Trust Level

The trust level *T*_TL_(*S*, *T*) was computed based on *T*_DL_(*S*, *T*) and *T*_IL_(*S*, *T*).

(8) $$\begin{array}{c} T_{\text{TL}}\left(S,T\right)=\alpha T_{\text{DL}}\left(S,T\right)+\beta T_{\text{IL}}\left(S,T\right), \end{array}$$

where *α* > *β* and *α* + *β* = 1.

The trust level was predicted based on direct trust level, number of fluctuations, and recommendation inconsistency.

### 3.6. Fuzzy-Based Trust Prediction Model

The fuzzy trust prediction model has three inputs, namely, direct trust value (*T*_DTV_(*S*, *T*)), number of fluctuations (*T*_*F*_), and recommendation inconsistency (*R*), and one output, trust level (*T*). The fuzzy membership functions for direct trust value are LOW (L), MEDIUM (M), and HIGH (H). The fuzzy membership functions for the number of fluctuations are LOW (L), MEDIUM (M), and HIGH (H). The fuzzy membership functions for the recommendation inconsistency are LOW (L) and HIGH (H). The fuzzy membership functions for the fuzzy output predicted trust are VERY LOW (VL), LOW (L), MEDIUM (M), HIGH (H), and VERY HIGH (VH). The rule bases of the evaluator are shown in Table 2. The bases of functions are chosen so that they result in optimal value of performance measures. To illustrate one rule, the first rule can be interpreted as, “If the direct trust value is LOW and number of fluctuations is LOW and recommendation inconsistency is LOW, the predicted trust is medium.” Similarly the other rules are framed.

## 4. Performance Analysis

The performance of FTPR protocol was evaluated using ns-2 simulator. The simulation parameters are listed in Table 3. We took a simulation area of 300 m × 300 m, with six hundred nodes placed at random. The transmission range was 45 m. IEEE 802.15.4 was the MAC layer protocol used to evaluate the performance of the proposed trust model under attack conditions.

### 4.1. Metrics

The performance of FTPR routing protocol was evaluated using the following metrics.

- *Packet Loss*. The total number of data packets lost legitimately or through malicious action without any notification.
- *Packet Delivery Ratio (PDR)*. The ratio of total number of data packets delivered to the total number of data packets sent.
- *Control Overhead*. The ratio of total number of control packets generated in the network to the total number of data packets received.
- *Energy Consumption*. The average energy consumed by each node during the given simulation time and expressed in Joules (J).
- *Network Lifetime*. Time taken for the energy of the first node to fall from 0.5 J to zero and expressed in seconds.
- *End-to-End Delay*. The delay experienced by the data packet during transmission from source to sink, including processing, queuing, and propagation delay.
- *Communication Overhead*. The total number of packets generated for trust establishment in the network. It included TREQ, TREP, acknowledgments, and warning and response packets.
- *Memory Consumption*. The total memory space exclusively used for trust derivation and representation, expressed in bits.

### 4.2. Simulation Results and Discussion

**Figure 3:**
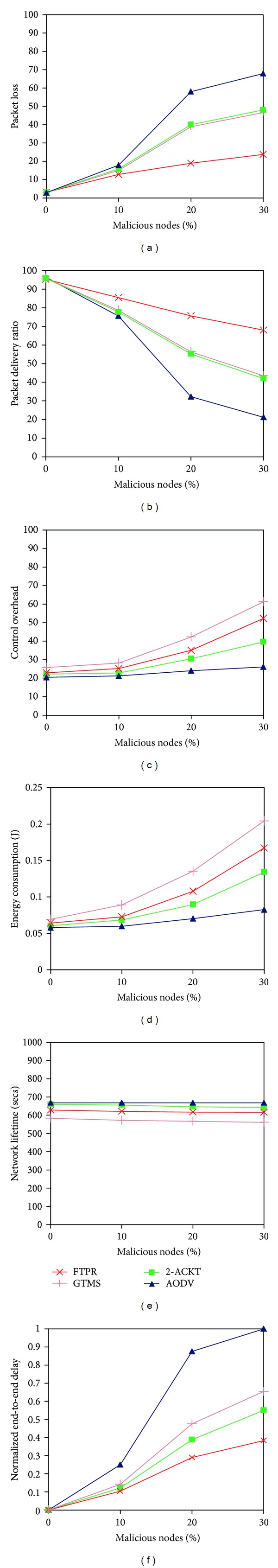
Performance comparison of FTPR, 2-ACKT, GTMS and AODV routing protocols under varying percentage of malicious attacks.

The performance of FTPR protocol was compared with 2-ACKT [17], GTMS [12], and AODV [23] protocols under varying number of malicious nodes as shown in Figure 3.

Among the total number of malicious nodes, 40 percent performed black hole attack, 30 percent performed on-off attack, 15 percent performed bad-mouthing attack, and 15 percent performed conflicting behavior attack. The FTPR, 2-ACKT, GTMS, and AODV routing protocols were tested against exactly the same scenario and connection pattern. The packet loss of FTPR, 2-ACKT, GTMS, and AODV protocols was plotted against varying percentage of malicious attacks as shown in Figure 3(a). The AODV is a traditional routing protocol which cannot thwart any malicious attacks and hence resulted in higher packet loss compared to FTPR, 2-ACKT, and GTMS. The GTMS and 2-ACKT were designed to thwart only black hole attack. The presence of on-off attack, bad-mouthing attack, and conflicting behavior attack resulted in higher packet loss in GTMS and 2-ACKT protocols. In GTMS and 2-ACKT, a node forwarded the data packets to its malicious neighbor until the trust level of that neighbor dropped below the *T*_TL_. But in FTPR, the node transmitted a packet to its next hop neighbor based on the predicted trust level and as a result, the packet loss in FTPR protocol is 43.53 percent and 45.24 percent lower than GTMS protocol and 2-ACKT protocol, respectively. As the malicious nodes were identified only based on direct trust in 2-ACKT, the packet loss was slightly higher when compared to GTMS. It has a positive effect on the PDR of FTPR protocol as shown in Figure 3(b). The PDR of FTPR routing protocol is augmented by 43.91 percent, 19.78 percent, and 18.18 percent when compared to AODV, 2-ACKT, and GTMS protocols, respectively.

In FTPR routing protocol, only direct observation was considered to compute trust in intracluster level and in intercluster level; the recommendations were collected only from the most interacted neighbors. In GTMS, the recommendations were considered both in the intracluster level and in the intercluster level. As the promiscuous mode of operation was not used for neighbor monitoring, the control overhead of FTPR protocol is 13.99 percent lower than the GTMS protocol as shown in the Figure 3(c). As the recommendations were not gathered, the control overhead of 2-ACKT protocol is 15.04 percent lower than FTPR.

The lower control overhead and the effective trust prediction mechanism in FTPR reduce the energy consumption by 17.26 percent when compared to GTMS protocol as shown in Figure 3(d). The energy consumption of GTMS is higher as the nodes use promiscuous mode for neighbor monitoring and also as the CHs use high powered transmitters to communicate with the BS. The simulation was performed with an initial energy of 0.5 J to calculate the network lifetime. The lower energy consumption improves the network lifetime of FTPR protocol by 8.72 percent higher than GTMS routing protocol as shown in Figure 3(e). Even though the control overhead of 2-ACKT and AODV was lower than FTPR, the presence of malicious nodes resulted in higher end-to-end delay in AODV, 2-ACKT, and GTMS as most of the data packets had not reached the destination as shown in Figure 3(f).

### 4.3. Theoretical Analysis

Let us assume that “*N*” is the total number of SNs in the network and let “*h*” be the average number of hops between a CM and the sink. For this analysis, we assumed that all nodes in the network wanted to communicate with the sink using “*h*” hops and did not have any prior trust relationship between their neighbors. In this section, the performance of FTPR protocol was compared with the GTMS [12] protocol in terms of communication overhead and memory consumption.

#### 4.3.1. Communication Overhead

In FTPR, when a node from *i*th cluster wants to communicate with the sink through its CH, the total number of acknowledgments generated for trust computation was 2(*h* − 1). Assuming a maximum of 30 percent malicious nodes, the maximum number of warning packets generated in the network was 0.3 × 3(*h* − 1) and the maximum number of response packet generated was 0.3 × 3(*h* − 1). Therefore, the communication overhead incurred by direct observation for one node to communicate with the sink is

(9) $$\begin{array}{c} 2\left(h-1\right)+\left(0.3\times 6\left(h-1\right)\right). \end{array}$$

The indirect observation was considered to compute trust between clusters. FTPR broadcasts one recommendation request and receives recommendation only from a set of neighbors and let σ be the number of received recommendations. Therefore, the communication overhead incurred by indirect observation for one CH to communicate with the sink is

(10) $$\begin{array}{c} 1+\sigma \left(h-2\right). \end{array}$$

So the total communication overhead incurred by one node to communicate with the sink is

(11) $$\begin{array}{c} 1+\sigma \left(h-2\right)+2\left(h-1\right)+\left(0.3\times 6\left(h-1\right)\right). \end{array}$$

When all the *N* nodes in the network wanted to communicate with the sink, the communication overhead is given by

(12) $$\begin{array}{c} C_{\text{FTPR}}=N\left[1+\sigma \left(h-2\right)+2\left(h-1\right)+\left(0.3\times 6\left(h-1\right)\right)\right]. \end{array}$$

As described in [19], the communication overhead of GTMS [12] protocol can be derived as

(13) $$\begin{array}{c} C_{\text{GTMS}}=2N\left(\delta +h-4\right). \end{array}$$

The communication overhead was plotted against the number of communicating nodes by setting *N* = 144 and *h* = 5 as shown in Table 4. The GTMS protocol with cluster size of 9, 12, and 18 nodes was represented as GTMS-9, GTMS-12, and GTMS-18, respectively. Communication overhead of GTMS protocol increases with cluster size as shown in Table 4, whereas the communication overhead of FTPR protocol was same throughout as it was not dependent on cluster size.

In GTMS, the recommendations were collected even in intracluster level and so, when the cluster size was large, more numbers of recommendations were received in GTMS protocol. As a result, the communication overhead of GTMS-18 is 17.9 percent higher than our proposed FTPR protocol.

In GTMS protocol, the CH employed a high power transmitter to directly communicate with the sink for requesting and gathering recommendations about the state of neighboring CHs. But in FTPR protocol, all the nodes used similar low power transmitters and communicated with the sink using a multihop link. The exchange of acknowledgments, warning and response packets in the multihop link increases the overhead of FTPR protocol. As a result, the communication overhead of GTMS-9 was 35.8 percent lower when compared to that of FTPR. Hence, FTPR is more suitable for large cluster sized homogenous WSNs.

#### 4.3.2. Memory Consumption

In FTPR, CMs and CHs maintained a transaction table to monitor and store the trust level of their neighbors. The fields in the transaction table and its memory size of CMs are shown in the Table 5. The node id occupied 2 bytes, number of successful transactions and number of failed transactions occupied 2 bytes each for each observation window present in the sliding time window, and trust level required 3 bits. Therefore, the memory required to store a record in the transaction table that represented the trust relationship with a neighbor was 2.375 + 4*n* bytes, where *n* is the number of observation windows.

The fields in the transaction table and its memory size of CHs are shown in Table 6. The CH contains 3 more additional fields than CM, namely, peer recommendations that occupy 3 bits and number of fluctuations and recommendation inconsistency occupying 4 bits each. Therefore, the memory required by a CH to store a record in the transaction table that represented the trust relationship with a neighbor was 3.75+4*n* bytes, where *n* is the number of observation windows.

The total size of the transaction table that represented the trust relationship between a CM and all its neighbors was

(14) $$\begin{array}{c} M_{\text{FTPR}(\text{CM})}=\left(2.375+4n\right)\left(\delta -1\right)\;\text{bytes}, \end{array}$$

where *δ* is the average size of cluster.

The total size of the transaction table that represented the trust relationship between a CH and all its neighbors was

(15) $$\begin{array}{c} M_{\text{FTPR}(\text{CH})}=\left(6.125+4n\right)\left(\frac{N}{\delta }-1\right)\;\text{bytes}, \end{array}$$

where *η*_av_ is the average number of CHs.

Therefore, the total memory consumed for the entire network which consisted of *N* number of CMs and *N*/*δ* number of CHs was

(16) MFTPR=(2.375+4n)(δ−1)N+(6.125+4n)(Nδ−1)(Nδ) bytes.

As described in [21], the memory consumption of GTMS [12] protocol can be derived as

(17) $$\begin{array}{c} M_{\text{GTMS}}=\left(6+4n\right)\left\{N\left(\delta -1\right)+\left(\frac{N}{\delta }\right)\left(\frac{N}{\delta }+\delta -2\right)\right\}\;\text{bytes}. \end{array}$$

The memory consumption for FTPR and GTMS protocols was plotted against the number of neighboring nodes and setting the size of the observation window *n* = 4 as shown in Table 7.

It was found that the memory consumption in FTPR protocol is 19.9 percent lower than the GTMS protocol. It was achieved due to the use of 3 bits to represent trust levels of the neighboring nodes in the transaction table and also direct trust was only considered in intercluster level.

## 5. Conclusions and Future Scope

In this paper, we proposed FTPR protocol to effectively thwart black hole attack, on-off attack, conflicting behavior attack, and bad-mouthing attack. It employed a fuzzy-based trust prediction model to predict the future behavior of a neighboring node based on its historical behavior, trust fluctuations, and recommendation inconsistency. It derived the trust based on the direct and indirect observations. It reduces the energy consumption significantly by avoiding the promiscuous mode of operation for direct trust derivation and by gathering recommendations only from a subset of neighbors for indirect trust derivation. The memory consumption is significantly reduced by representing 8 bit trust values as 3 bit trust levels.

By considering the historical behavior of node using sliding time window scheme, the on-off attack was identified and eliminated. The bad-mouthing attack was avoided effectively by eliminating outliers from the received recommendations. The conflicting behavior was thwarted by considering recommendation inconsistency in the fuzzy-based trust prediction. The novel trust prediction model significantly improved the packet delivery ratio in the network. As the recommendations were received only from a subset of neighbors, there was a significant reduction in control overhead. Theoretical and simulation results of FTPR protocol demonstrate higher packet delivery ratio, lower end-to-end delay, higher network lifetime, and lower memory consumption than the traditional and existing trust-based routing schemes. The limitation of this research work was that the nodes were assumed to have unique identity which is not suitable for some applications. So, we plan to design a trust-based routing protocol for applications that require anonymous identity in WSNs.

## Figures and Tables

**Algorithm 1:**
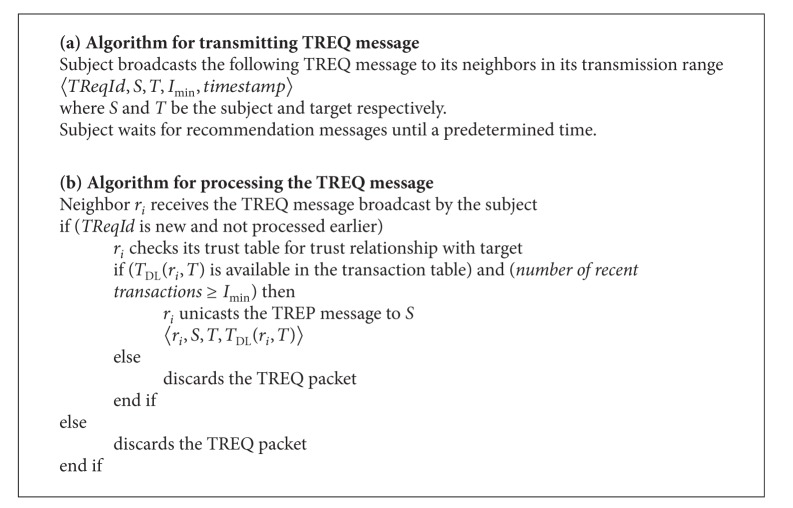
Algorithm for processing TREQ and TREP.

**Table 1:** Status field in response packet.

Status	Description
0	Link failure and not ready to forward
1	Link failure but now ready to forward
2	Insufficient resource and not ready to forward
3	Insufficient resource but now ready to forward
4	Non co-operative neighbor and no alternate path
5	Non co-operative neighbor but new alternate path available

**Table 2:** Fuzzy rule base.

Rule number	Direct trust value	Number of fluctuations	Recommendation inconsistency	Predicted trust
1	L	L	L	M
2	L	L	H	L
3	L	M	L	M
4	L	M	H	L
5	L	H	L	VL
6	L	H	H	VL
7	M	L	L	H
8	M	L	H	M
9	M	M	L	M
10	M	M	H	L
11	M	H	L	M
12	M	H	H	L
13	H	L	L	VH
14	H	L	H	M
15	H	M	L	H
16	H	M	H	M
17	H	H	L	H
18	H	H	H	L

**Table 3:** Simulation parameters.

Simulation time	800 secs
Simulation area	300 m × 300 m
Number of nodes	600
Frequency of operation	2.4 GHz
Node placement	Random
Transmission range	45 m
Propagation model	Two-ray
Movement model	Static
Traffic type	CBR (UDP)
Packet size	50 bytes
Packet interval	10 secs
Maximum number of malicious nodes	180
Type of attack	Black hole, on-off attack, conflicting behavior attack, and bad-mouthing attack
Initial energy	2 Joules
T_TL_	4

**Table 4:** Communication overhead of FTPR and GTMS.

Number of communicating nodes	Communication overhead			
	GTMS-9	GTMS-12	GTMS-18	FTPR
36	720	936	1368	1123
72	1440	1872	2736	2246
108	2160	2808	4104	3369
144	2880	3744	5472	4492

**Table 5:** FTPR CM trust table.

Node id	Number of successful transactions			Number of failed transactions			Trust level
	*w*_1_	⋯	*w*_*n*_	*w*_1_	⋯	*w*_*n*_	
2 bytes	2 bytes		2 bytes	2 bytes		2 bytes	3 bits

**Table 6:** FTPR CH trust table.

Node id		2 bytes
Number of recent transactions		2 bytes

Number of successful transactions	*w*_1_	2 bytes
	⋮	
	*w*_*n*_	2 bytes

Number of failed transactions	*w*_1_	2 bytes
	⋮	
	*w*_*n*_	2 bytes

Trust level		3 bits

Peer recommendations		3 bits

Number of fluctuations		4 bits

Recommendation inconsistency		4 bits

Predicted trust		3 bits

**Table 7:** Memory consumption of FTPR and GTMS.

Number of neighboring nodes	Memory consumption (bytes)	
	GTMS	FTPR
9	26478	25998
12	32026.5	31762.5
16	41283	41139
18	46221	46109
